# The use of cloud based machine learning to predict outcome in intracerebral haemorrhage without explicit programming expertise

**DOI:** 10.1007/s10143-024-03115-3

**Published:** 2024-12-03

**Authors:** Ajay Hegde, Deepu Vijaysenan, Pitchaiah Mandava, Girish Menon

**Affiliations:** 1 Neurosurgery, Kasturba Medical College, Manipal Academy of Higher Education, 576104 Manipal, India; 2 Neurosurgery, Manipal Hospitals, Bangalore, India; 3 Department of Electronics and Communication Engineering, National Institute of Technology, Surathkal, Karnataka India; 4 Baylor College of Medicine, Houston, TX USA; 5 Neurosurgery, Kasturba Medical College, Manipal Academy of Higher Education, Manipal, 576104 India

**Keywords:** Intracerebral haemorrhage, Outcome, Machine learning, Google AutoML

## Abstract

Machine Learning (ML) techniques require novel computer programming skills along with clinical domain knowledge to produce a useful model. We demonstrate the use of a cloud-based ML tool that does not require any programming expertise to develop, validate and deploy a prognostic model for Intracerebral Haemorrhage (ICH). The data of patients admitted with Spontaneous Intracerebral haemorrhage from January 2015 to December 2019 was accessed from our prospectively maintained hospital stroke registry. 80% of the dataset was used for training, 10% for validation, and 10% for testing. Seventeen input variables were used to predict the dichotomized outcomes (Good outcome mRS 0–3/ Bad outcome mRS 4–6), using machine learning (ML) and logistic regression (LR) models. The two different approaches were evaluated using Area Under the Curve (AUC) for Receiver Operating Characteristic (ROC), Precision recall and accuracy. Our data set comprised of a cohort of 1000 patients. The data was split 8:1 for training & testing respectively. The AUC ROC of the ML model was 0.86 with an accuracy of 75.7%. With LR AUC ROC was 0.74 with an accuracy of 73.8%. Feature importance chart showed that Glasgow coma score (GCS) at presentation had the highest relative importance, followed by hematoma volume and age in both approaches. Machine learning models perform better when compared to logistic regression. Models can be developed by clinicians possessing domain expertise and no programming experience using cloud based tools. The models so developed lend themselves to be incorporated into clinical workflow.

**Supplementary Information:**

The online version contains supplementary material available at 10.1007/s10143-024-03115-3.

## Introduction

The adoption of human visual processing inspired by machine / deep learning (ML/DL) approaches to medicine has opened new possibilities. Novel Machine learning algorithms inspired by human visual processing [1] allow computers to identify patterns in data and build models without having explicit pre-programmed rules and models. There are broadly two main types of ML techniques used: supervised and unsupervised [2]. The former trains from a set of data labelled by humans as one class or other to predict outcomes [3]. Unsupervised learning analyses un-labelled data and the machine tries to find relations between the data set by comparing similarities within them without human input. A balanced hybrid learning technique called semi-supervised exists, where there are only a few labelled data in a larger pool of un-labelled data.

ML has been used by Neurosurgeons for presurgical planning, intraoperative guidance, neurophysiological monitoring, and delineation of the intracerebral tumours [4–10]. In seven studies that compared ML models with classical logistic regression, ML models performed significantly better [4]. On the other hand, predictions of long term outcome in a neurosurgical emergency, has not been adequately tackled by ML approaches.

Intracerebral haemorrhage (ICH), a neurosurgical emergency, with high morbidity and mortality is the second most common cause of stroke accounting for 15–30% of all strokes [11, 12]. Optimal treatment regimens and standardised guidelines are yet to evolve and no universally adopted strategies have been seen to work [13]. ICH is predominantly a disease of the elderly but is also seen in younger adults of the working age group in the Asian population [14–17]. Considering this global problem, several prognostic scoring systems have been developed based on predominantly Caucasian populations. Amongst the many regressions based models, the ICH score is the most validated and widely adapted score used to predict 30-day mortality [18]. Despite several decades’ long history of regression-based modelling, a clinicians prediction of outcomes for an individual patient was considered superior to any existing regression-based models [19]. However, there has been a paucity of attempts to apply ML algorithms to predict outcome and mortality in ICH and compare them against a logistic regression based model.

In a global survey, one of the main reasons for the relative absence of the application of ML in Neurosurgery, was the lack of resources and programming skills to develop and apply a ML model [20]. We attempted to address this issue of lack of programming expertise by demonstrating how an ML tool can be developed by a Neurosurgical clinician domain expert-led team with minimal resources in a cost-conscious economy like India. Several cloud-based ML user interfaces are available that enable users with a limited programming language but with sufficient domain knowledge to conceptualize a high-level model, train, and validate models specific to their needs. Models so developed can be exported and integrated into clinical workflow tools. We used Google AutoML© Tables, which is a supervised deep learning service. It uses tabular (structured) data to train an ML model to make predictions on new data. We compared the results of ML based prediction to traditional logistic regression.

## Materials and methods

### Patient data

We accessed data of patients admitted with spontaneous ICH from our prospectively maintained hospital Stroke registry between January 2015 and December 2019 after necessary regulatory approvals. Demographic data, baseline clinical characteristics, Glasgow Coma Score (GCS:3–15), blood pressure, comorbidities, blood glucose on admission, radiological data including volume of bleed, computed using abc/2 formula, location, intraventricular extension, laterality, and hydrocephalus were recorded. Outcome at 90 days was expressed using the six-point Modified Ranking Scale. When a patient failed to visit the clinic, mRS assessed by a trained physician employing an established telephonic questionnaire was recorded [21]. The 90-day outcome, which was the output of the model, was dichotomized mRS with the cut-off value of mRS 3 (Good outcome: mRS 0–3 and bad outcome of mRS 4–6). This dichotomous cut-point was chosen since this cut-point is reported to have the least information loss [22]. Continuous and nominal variables did not have any missing values, while ordinal variables with missing values were allowed to be included in the model.

The data set was split manually in the ratio 0.9 by the *‘dyplr’* package for R such that the test data for both platforms remained identical.

### Machine learning Model - AutoML© Tables

A total of 1,000 entries were used as this was the minimum required for Google Cloud ML. (Supplement Fig. 1). Data splitting was performed manually with test data being labelled as “TEST”.

Cramér’s V correlation statistic between input variables and the target column (outcome) was computed in the first stage to help decide the significance of each variable on the outcome (Supplement Table 1). This ranges from zero to one, where zero indicates no correlation and one indicates perfect correlation. A low correlation suggests that the column can be excluded from the model without much performance penalty.

The model was evaluated based on the following computed parameters. AUC PR (area under the curve, precision recall) is the area under the precision-recall curve and AUC ROC (area under the curve, receiver operating characteristic) is the area under the receiver operating curve. Both these have values ranging from zero to one with a higher value indicating a more precise model. The accuracy/precision of the model is the percentage of correct classification predictions produced by the model. Log loss is the cross-entropy between the model predictions and the target values. It ranges from zero to infinity, where a lower value indicates a higher-quality model. F1 score is the harmonic mean of precision and recall. F1 is a useful metric if you are looking for a balance between precision and recall and there is an uneven class distribution. Recall or true positive rate is the fraction of rows with this label that the model correctly predicted, and the false positive rate is the fraction of rows predicted by the model to be the target label but is not. A Confusion/Error Matrix of predicted labels vs. *True* labels also termed as *ground truth* was created. The relative importance of each variable was computed using a feature importance chart which was determined by how much the prediction varies when the value of the column changes. These scores are normalized to sum to one. A higher variance in the prediction indicates the greater importance of the variable. The generated model could be exported in a Docker container as a TensorFlow package for easy deployment.

### Logistic regression model

The data set was processed using RStudio for Windows V2022.02. The *‘glm’* function was then used to build a logistic model. The results of this model were then used to predict responses in the test data and a confusion matrix was generated. ROC curve was then plotted based on the prediction and performance of the model using the *‘plot’* function. The model was evaluated using the Confusion matrix, AUC (Area under the curve), ROC (Receiver operating characteristics) curve, precision recall and accuracy.

## Results

A total of 1,000 patients admitted to our centre with ICH during the study period were analyzed. Outcome (Good/Bad) defined by mRS was predicted based on seventeen admission variables. A snapshot of the demographic, clinical and radiological parameters with outcome of our dataset are listed in Supplement Table 2. The input variables were Categorical − 10 and Numeric 7. Continuous variables had no missing values. Smoking, Antiplatelet use, alcohol intake and diabetes mellitus had null values, which were allowed to be used in the training models. (Table 1). The data was split in the ratio 9:1, with 889 cases used for training and validation and 111 for testing.

**Table 1:** Demographic, clinical, radiological distribution of our cohort

Parameter	*n* = 1000
Age (years)	58.34±12.75
Sex (M: F)	701:299
Hypertension	577 (57.7%)
Diabetes Mellitus	265 (26.5%)
Alcohol consumption	295 (29.5%)
Smoking	148 (14.8%)
Antiplatelet medications	97 (9.7%)
Heart Rate	81.91±16.37
Systolic Blood Pressure	174.39±57.26
Diastolic Blood Pressure	99.17±14.26
GCS	12 ± 4
Blood Glucose	161.30±68.32
Hematoma volume	21.67±19.18
Laterality (Right: Left: Midline: Bilateral)	472: 463: 57: 8
Location (Basal Ganglia: Thalamus: Lobar: Cerebellar: Brainstem: Primary IVH)	526: 219: 122: 67: 35: 31
Intraventricular extension	465 (46.5%)
Hydrocephalus	247 (24.7%)
Good Outcome	473 (47.3%)
Poor Outcome	527 (52.7%)

## Machine learning model

Cramér’s V correlation statistic was evaluated and GCS on admission had the highest value at 0.6, followed by a volume of haematoma 0.57. The least value was for sex (0.129) (Supplement Table 1). The model was manually split to assign test values. AutoML© tables analyzed the data for a total of 0.91 node hours to produce results using theAdanet Autoemsembler after running iterations of Adanet and gradient boosted decision trees (GBDT). The model had one hidden layer of size 116 and a drop out of 0.319. (Supplement Table 2) The model was optimized for AUC ROC and Accuracy is based on a score threshold of 0.5. The AUC PR and AUC ROC for the model were 0.82 and 0.86 respectively, with an accuracy of 75.7%.

**Fig. 1:**
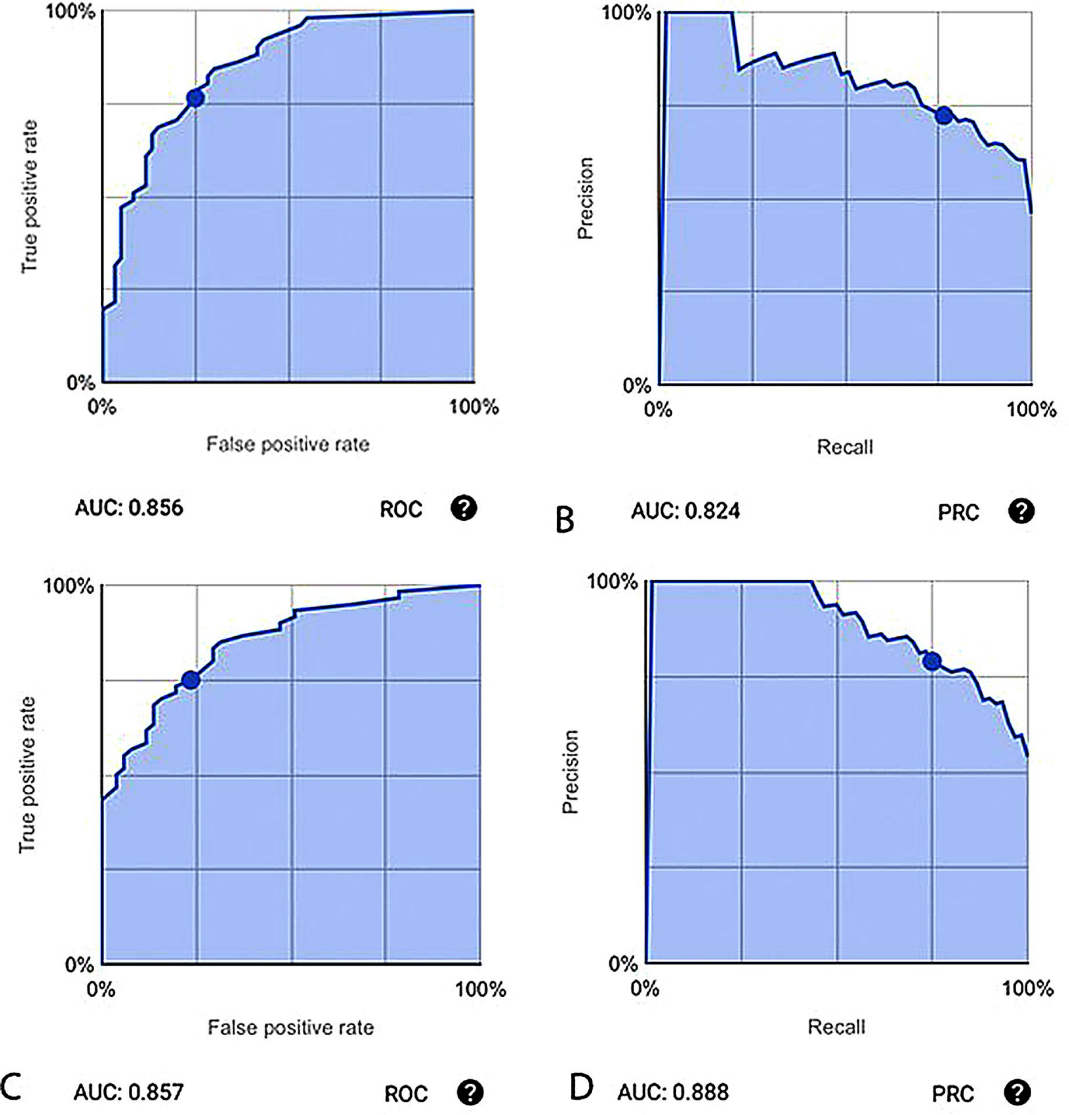
Area under the curve for machine learning model

For prediction of a good outcome, the F1 score was 0.743 with an accuracy of 75.7% (84/111), the precision of 72.2% (39/54), true positive rate (recall) of 76.5% (39/51),

and false positive rate of 0.25 (15/60) (Fig. 1A&B). For prediction of poor outcome, the F1 score was 0.769 with an accuracy of 75.7% (84/111), the precision of 78.9% (45/57), true positive rate (recall) of 75% (45/60), and false positive rate of 0.235 (12/51) (Fig. 1C&D). The confusion matrix generated is described in Table 2, with a false negative rate 23.5% (12/51) and false positive rate of 25% (15/60).

**Fig. 2:**
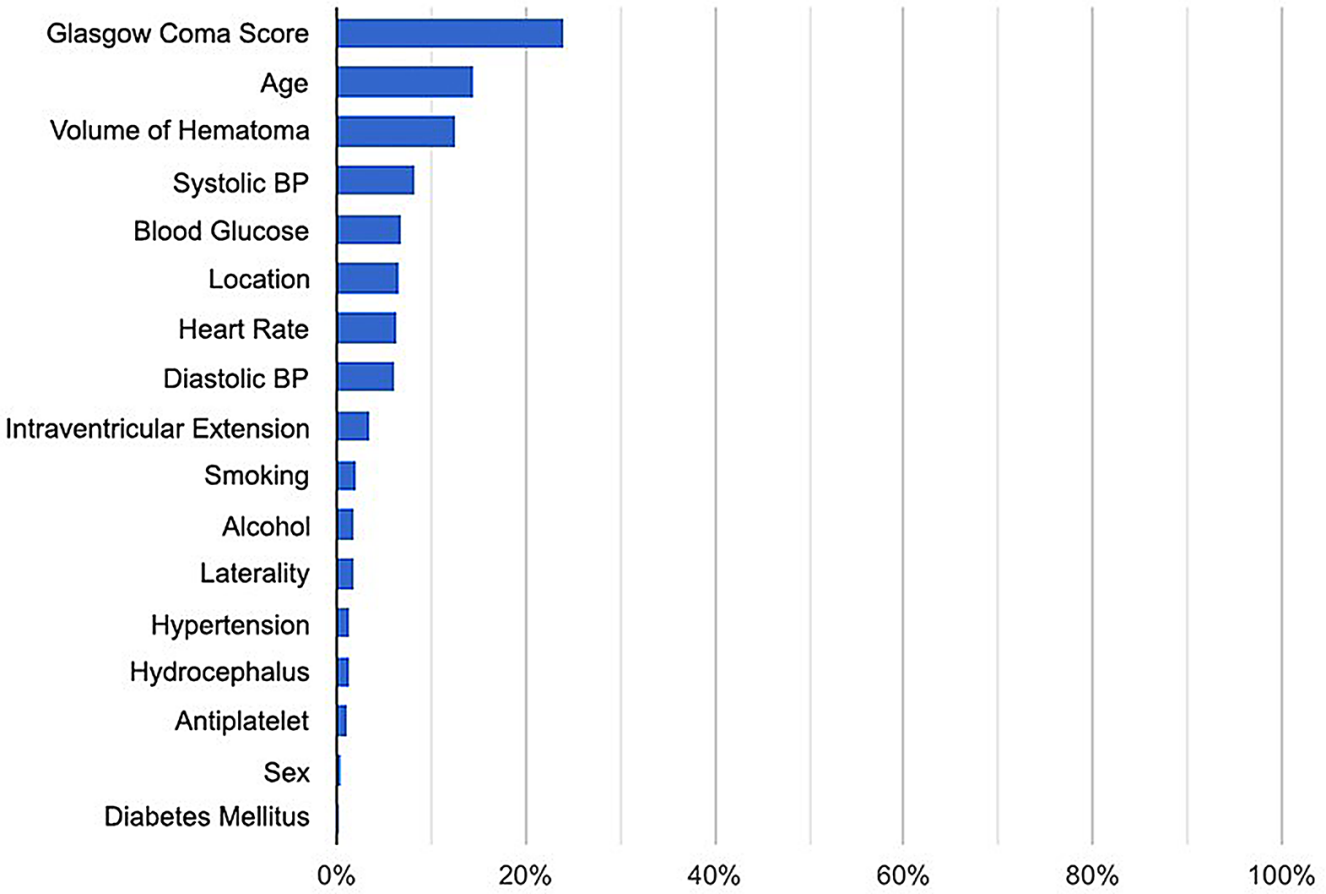
Feature importance chart

**Table 2:** Confusion matrix for ML and LR models

True Labels	Predicted Labels			
	Machine Learning (ML)		Logistic Regression (LR)	
	Good	Poor	Good	Poor
Good	39/51 (76.5%)	12/51 (23.5%)	39/51 (76.5%)	12/51 (23.5%)
Poor	15/60 (25%)	45/60 (75%)	17/60 (28.3%)	43/60 (71.7%)

The feature importance chart showed that GCS has the highest relative importance at 24.16, followed by age and volume of hematoma. Presence of diabetes mellitus and sex had the lowest importance in our cohort (Fig. 2).

## Regression model

**Fig. 3:**
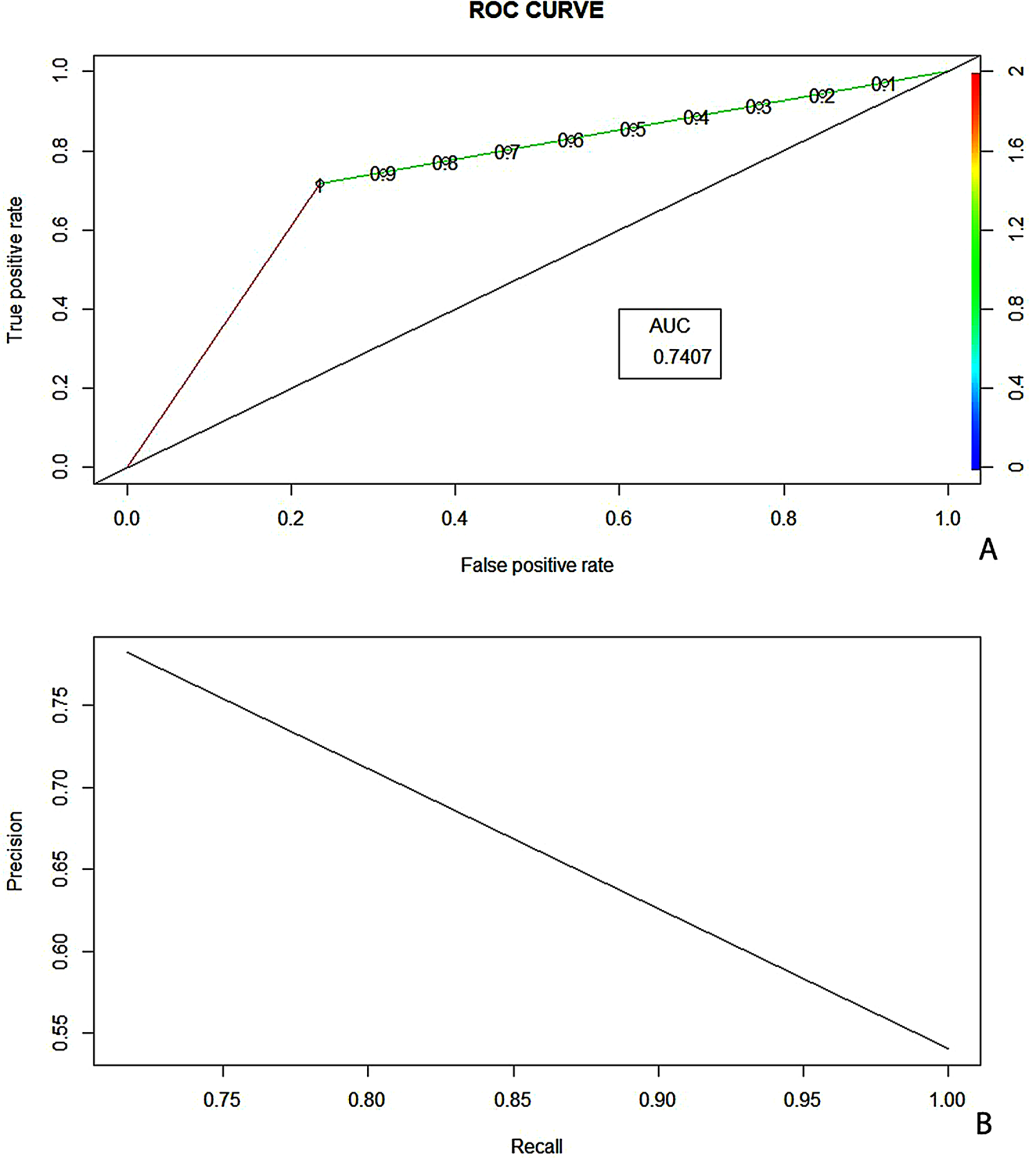
Area under the curve for logistic regression model

Logistic regression was run on the training data. Age, GCS on admission, Volume of hematoma, Location (Lobar and Cerebellum), total white cell count and haemoglobin were found to be significant factors (*P* < 0.05) in the prediction of dichotomised outcome. (Table 3) Confusion matrix was generated, (Table 2) with a false negative rate of 23.5% (12/51) and false positive rate of 28.3% (17/60). Multivariate analysis using logistic regression had the highest Z value for GCS followed by age and volume of hematoma. (Table 3) Area under curve for ROC to measure performance was 0.74 and precision recall was 0.78. (Fig. 3A& B) with an accuracy of 73.8%. A comparison of results for ML and LR models are in Table 4.

**Table 3:** Logistic regression results

Parameter		Estimate	Std. error	z value	Pr(>|z|)
(Intercept)		2.985E + 01	1.543E + 03	0.019	0.98457
*Age*		*6.307E-02*	*8.301E-03*	*7.598*	3.00e-14 ***
Sex	Male	-2.807E-03	2.153E-01	-0.013	0.9896
Side	Left	-1.430E + 01	5.127E + 02	-0.028	0.97775
	Midline	-1.496E + 01	5.127E + 02	-0.029	0.97673
	Right	-1.427E + 01	5.127E + 02	-0.028	0.9778
Hypertension	Yes	1.172E-01	1.898E-01	0.617	0.53709
Diabetes Mellitus	No	-1.617E + 01	1.455E + 03	-0.011	0.99114
	Yes	-1.619E + 01	1.455E + 03	-0.011	0.99113
Smoking	Yes	3.380E-01	4.617E-01	0.732	0.4641
	No	-1.777E-01	3.886E-01	-0.457	0.6474
Alcohol	No	-9.083E-02	3.536E-01	-0.257	0.79725
	Yes	-1.020E-01	3.939E-01	-0.259	0.79565
Antiplatelet	No	-1.341E + 00	1.035E + 00	-1.296	0.19493
	Yes	-7.673E-01	1.075E + 00	-0.714	0.47553
*Heart Rate*		*1.211E-02*	*6.001E-03*	*2.017*	0.04366 *
Systolic BP		-2.615E-03	3.002E-03	-0.871	0.38379
Diastolic BP		6.996E-03	8.061E-03	0.868	0.38545
*Glasgow Coma Score*		*-3.493E-01*	*3.844E-02*	*-9.088*	< 2e-16 ***
*Blood Glucose*		*2.876E-03*	*1.569E-03*	*1.833*	*0.06681.*
Intraventricular Extension		3.364E-01	2.341E-01	1.437	0.15069
*Volume of Hematoma*		*4.059E-02*	*8.999E-03*	*4.511*	6.46e-06 ***
Hydrocephalus		3.225E-01	2.857E-01	1.129	0.25898
Location	Brainstem	1.283E + 00	1.179E + 00	1.088	0.27648
	*Cerebellar*	*-1.186E + 00*	*4.350E-01*	*-2.725*	0.00642 **
	*Lobar*	*-8.810E-01*	*2.755E-01*	*-3.198*	0.00138 **
	Primary IVH	-1.162E + 00	1.110E + 00	-1.047	0.29512
	Thalamus	-4.024E-01	2.919E-01	-1.378	0.16808

Signif. Codes: 0 ‘***’ 0.001 ‘**’ 0.01 ‘*’ 0.05 ‘.’ 0.1 ‘ ’ 1

**Table 4:** Comparison between the two models

Measurement	Deep learning model	Logistic regression model
AUC ROC	0.86	0.74
AUC PR	0.82	0.78
Accuracy	75.7%	73.8%

## Discussion

There has been a resurgence of interest in the field of artificial intelligence (AI) due to the adoption of novel ML/DL algorithms. The expanded access to computing power provided by Graphics Processing Unit (GPU) technology and healthcare data especially in low and middle-income countries has sparked an increasing interest in AI due to the perceived failures of traditional regression-based models vis-à-vis an expert prediction [23, 24]. A meta-analysis of ML methods has shown that ML prediction outperforms logistic regression models as well as established prognostic indices by overcoming possible human error and bias. ML models have predicted neurosurgical outcome with a high accuracy of 94.5% (interquartile range [IQR] 87-95%; range 63-98%), and an AUC of 0.84 (IQR 0.82–0.88; range 0.71–0.96) [4, 25]. AI has the ability to learn, self-correct and improve its accuracy based on feedback, thus outperforming statistical analysis [23]. It has been successfully applied to predict outcomes in traumatic brain injury and ischemic stroke [26, 27]. 

Few studies have applied ML to predict outcomes in ICH. In a study by Wang et al. [28] with 333 patients, a random forest model provided an overall accuracy of 83.9%, with a sensitivity of 72.5%, specificity of 90.6%, and AUC of 0.917 in predicting six months’ outcome after ICH. Lin et al. applied AAN and Random forest models to the Taiwan National stroke registry with 4495 ICH patients and achieved a ROC of 0.95. The high precision of this model has been attributed to high-quality cleaned data and standardized outcome data [29]. Similar practises were followed in capturing data from our stroke registry. It is known that larger datasets with more variables performed better.

We restricted our model to seventeen selected variables that could be recorded at the first interaction with the patient even in semi-urban and rural areas. We also compared prediction of ML based with logistic regression-based prediction. The features having highest influence on the outcome selected by our ML and LR models were similar with GCS on admission, Age and volume of hematoma occupying the top three spots. Systolic Blood pressure and blood glucose on admission followed the above three features in the ML model, whereas they did not appear to be significant in predicting outcome in the LR model. In the LR mode, heart rate and location of bleed were significant factors predicting outcome. Our results show that ML based models generated on the automated cloud based platforms outperforms regression-based model marginally if not significant better. It is able to predict poor outcome better in our test cohort, thereby improving the accuracy of the analysis. AUC ROC and PR were better by 0.12 and 0.4 respectively. Accuracy in the ML model was better by 1.9%. AUC is a better measure of a model as it accounts for confidence of the class prediction [30]. The ROC of our ML model was 0.86 which makes it an excellent model to predict outcome in comparison to the to the LR model ROC 0.74.

There have been instances in other areas of clinical medicine where regression based models were superior to several different AI based modelling techniques [31]. It has been suggested that when the number of observations and number of variables are large, ML may perform better [32]. Conversely logistic regression out-performs when there are few numbers of variables albeit with high degree of linear relationship between the outcome variable and independent variables. Acquiring more data and clinical cases comes at a cost and is challenging in relatively rare diseases. Pooling data from different clinical centers increases the cost of cleaning the data and verifying methods of acquiring data are uniform across centers. Although ML based models are known to produce better results, it is not without caveats. They have suffered from what is often referred to as a “reproducibility crisis” [33]. The same data set when trained multiple times produced different outputs with different ROC curves. While the variation was not significant, it does not happen in LR based models. The challenges we discuss are equally applicable to the cloud-based platform outlined in our paper.

Most ML programs in medicine are developed based on only one architecture, due to time, skill, and cost issues. The advantage of AutoML© tables is that it runs the data through five different architectures (Linear, Feedforward Neural Network, Gradient Boosted Decision Tree, AdaNet, and ensembles of these models) and chooses the best model. This is a significant advantage without having to serially iterate over different model architectures. Our model was built on the AdaNet Autoensembler, which is a type of machine learning model for automatically learning high-quality ensembles with minimal expert intervention [34, 35]. Ensemble techniques create multiple models and then combine them to produce improved results which are more accurate than a single model. AdaNet combines two orthogonal ensembling paradigms [36] parallel and sequential. Together, these form the axes of the adaptive search space that the framework iteratively explores for an optimal ensemble [35]. The main advantage of ensemble models over traditional ML methods is that it outperforms the latter in several cases, particularly when learning from large datasets [29, 36]. 

The uniqueness in our model and implementation is of a clinician-driven development and machine learning-based outcome prediction model. Cloud based (Google AutoML©) with its easy interface and comprehensive guides makes this possible. While most models are developed by data scientists and teams with an extensive engineering background, we demonstrate that promise of AI that can be harnessed by physicians with no programming knowledge by adopting off the shelf available user interfaces. Any clinical team with high-quality data can harness Google’s state of the art prediction algorithms to improve patient care in their domain.

### Limitations

The entire process being automated, does not allow the user to choose the appropriate machine learning architecture. The data used in this study included patients from a single tertiary medical college hospital in southern India. The findings of this study must be validated in a different region with a more ethnically diverse patient population. ML-based models can produce different results with each training session on the same dataset, leading to reproducibility issues that are not present in linear regression models.

## Conclusion

Machine learning platforms can provide robust and accurate predictions in neurological illness with better accuracy when compared to logistic regression models. We demonstrate the ability to create and test a successful ML model designed by the Google Cloud AutoML© platform. The superiority of ML based models may be overestimated to statistical models. The strength of this system lies in its simplicity and independent deployment without coding knowledge.

## Electronic supplementary material

Below is the link to the electronic supplementary material.

Supplementary Material 1

## Data Availability

No datasets were generated or analysed during the current study.
