# Supplementary material for: The use of cloud based machine learning to predict outcome in intracerebral haemorrhage without explicit programming expertise
Source: Neurosurg Rev. 2024 Dec 3;47(1):883. doi: 10.1007/s10143-024-03115-3 (PMC11614922; doi:10.1007/s10143-024-03115-3)
Supplement: Supplementary file 1 — Supplementary Material 1 [file 10143_2024_3115_MOESM1_ESM.docx]

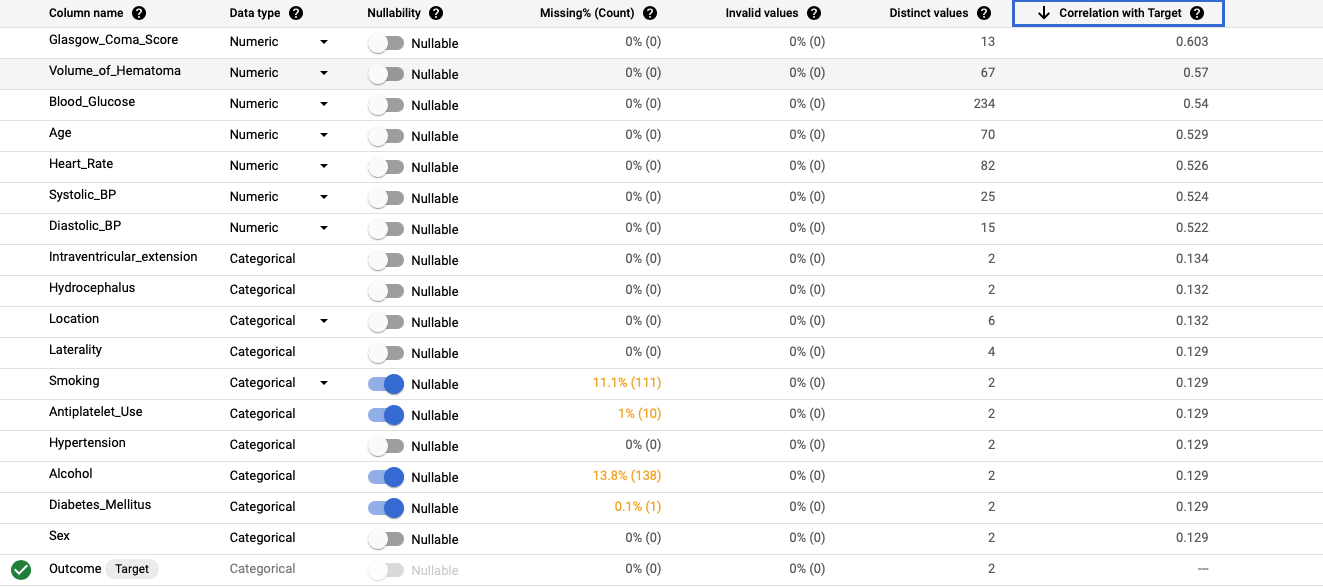


Supplement Table 1: Data used for deep learning model with Cramér's V correlation statistic.

| { |
| --- |
| "insertId": "crr01tfu2cf9k", |
| "jsonPayload": { |
| "modelParameters": [ |
| { |
| "hyperparameters": { |
| "Dropout": 0.3198576006971788, |
| "Hidden layer size": "116", |
| "Center bias": "False", |
| "L2 regularization strength": 0.0009249597028673168, |
| "Number of hidden layers": "1", |
| "Model type": "AdaNet AutoEnsembler", |
| "L2 shrinkage regularization strength": 1.004814665233164e-8, |
| "L1 regularization strength": 0.000024074081236934, |
| "Boosting iterations": "1", |
| "Growing mode": "layer", |
| "Max tree depth": 7, |
| "Tree complexity": "1" |
| } |
| } |
| ], |
| "@type": "type.googleapis.com/google.cloud.automl.master.TablesModelStructure" |
| }, |
| "resource": { |
| "type": "cloudml_job", |
| "labels": { |
| "project_id": "direct-obelisk-319309", |
| "job_id": "TBL2393942477900873728", |
| "region": "us-central1" |
| } |
| }, |
| "timestamp": "2022-04-22T16:56:57.289198298Z", |
| "severity": "INFO", |
| "labels": { |
| "log_type": "automl_tables" |
| }, |
| "logName": "projects/direct-obelisk-319309/logs/automl.googleapis.com%2Fmodel", |
| "receiveTimestamp": "2022-04-22T16:56:57.289198298Z" |
| } |
|  |

Table 2: Google AutoML Tables Model architecture.


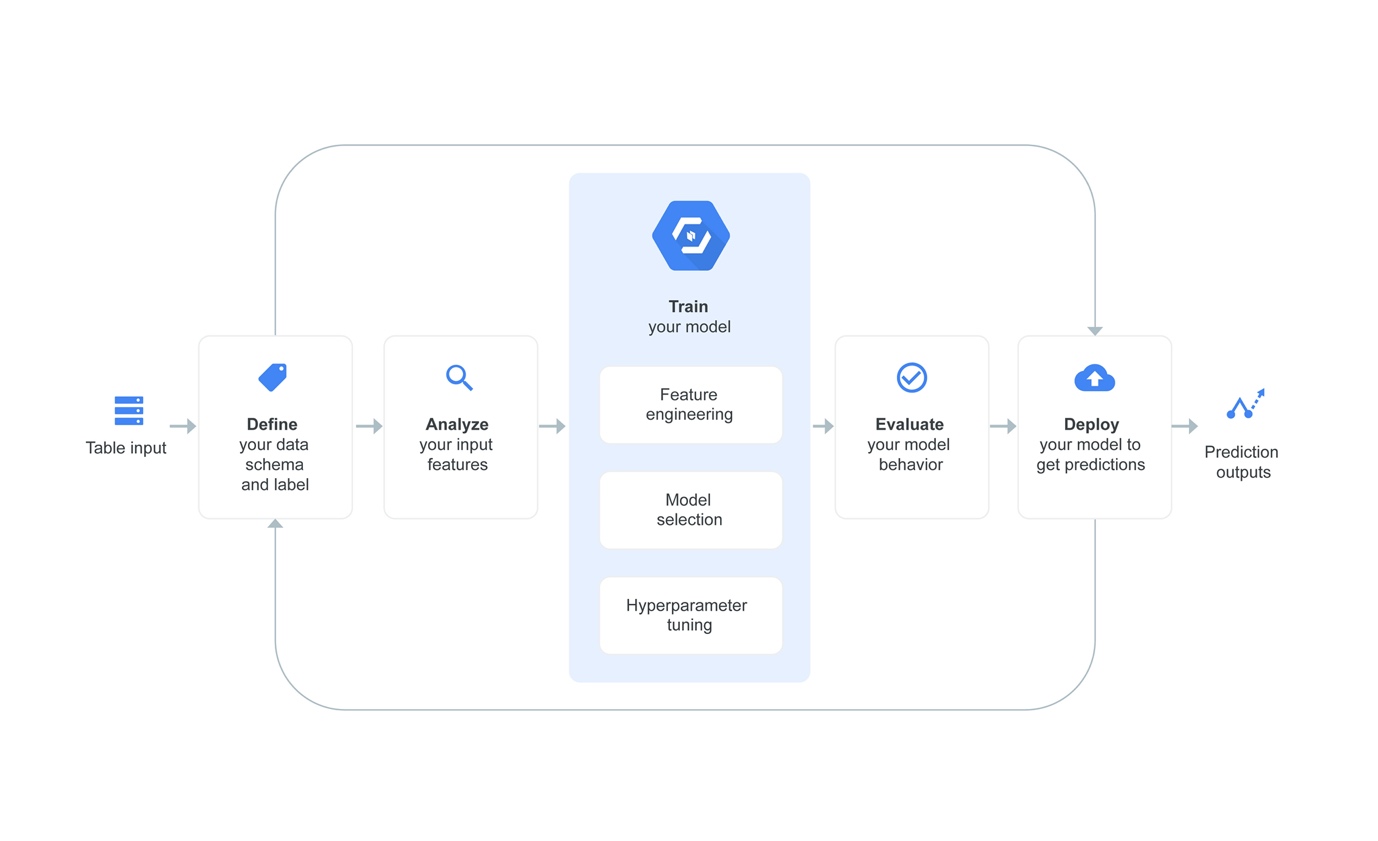


Supplement Figure 1: Steps of building a machine learning algorithm using Google Cloud. Source: <https://cloud.google.com>
